# Cortical Processing of Binaural Cues as Shown by EEG Responses to Random-Chord Stereograms

**DOI:** 10.1007/s10162-021-00820-4

**Published:** 2021-12-13

**Authors:** Henri Pöntynen, Nelli Salminen

**Affiliations:** Aalto Acoustics Lab, Department of Signal Processing and Acoustics, School of Electrical Engineering, Aalto University, Espoo, Finland

**Keywords:** Electroencephalography, Random-chord stereogram, Interaural envelope correlation, Binaural hearing, Auditory scene analysis

## Abstract

Spatial hearing facilitates the perceptual organization of complex soundscapes into accurate mental representations of sound sources in the environment. Yet, the role of binaural cues in auditory scene analysis (ASA) has received relatively little attention in recent neuroscientific studies employing novel, spectro-temporally complex stimuli. This may be because a stimulation paradigm that provides binaurally derived grouping cues of sufficient spectro-temporal complexity has not yet been established for neuroscientific ASA experiments. Random-chord stereograms (RCS) are a class of auditory stimuli that exploit spectro-temporal variations in the interaural envelope correlation of noise-like sounds with interaurally coherent fine structure; they evoke salient auditory percepts that emerge only under binaural listening. Here, our aim was to assess the usability of the RCS paradigm for indexing binaural processing in the human brain. To this end, we recorded EEG responses to RCS stimuli from 12 normal-hearing subjects. The stimuli consisted of an initial 3-s noise segment with interaurally uncorrelated envelopes, followed by another 3-s segment, where envelope correlation was modulated periodically according to the RCS paradigm. Modulations were applied either across the entire stimulus bandwidth (wideband stimuli) or in temporally shifting frequency bands (ripple stimulus). Event-related potentials and inter-trial phase coherence analyses of the EEG responses showed that the introduction of the 3- or 5-Hz wideband modulations produced a prominent change-onset complex and ongoing synchronized responses to the RCS modulations. In contrast, the ripple stimulus elicited a change-onset response but no response to ongoing RCS modulation. Frequency-domain analyses revealed increased spectral power at the fundamental frequency and the first harmonic of wideband RCS modulations. RCS stimulation yields robust EEG measures of binaurally driven auditory reorganization and has potential to provide a flexible stimulation paradigm suitable for isolating binaural effects in ASA experiments.

## Introduction

The auditory system faces the ill-posed scene analysis problem of having to separate sound mixtures arriving at the ears into behaviorally useful information about the identities and locations of sound sources in the environment (Bregman 1994). The apparent ease with which the sense of hearing accomplishes this task implies that systematic computational principles facilitate the process. Many salient scene analysis cues are accessible monaurally (Bregman 1994; Darwin 1997; Grimault et al. 2002; Moore and Gockel 2002; Carlyon 2004; Alain 2007; Snyder and Alain 2007; Shamma and Micheyl 2010; Moore and Gockel 2012; Simon 2015), and evidence for auditory perceptual organization has been found already in the cochlear nucleus (Pressnitzer et al. 2008). In addition, binaural hearing facilitates scene analysis and yields a significant advantage in various laboratory listening tasks benefiting from perceptual segregation of concurrent sound sources, as well as in behaviorally relevant scenarios such as “cocktail party” listening (Cherry 1953; Cherry and Taylor 1954; Arbogast and Kidd 2000; Bronkhorst 2000; Brungart 2001; Brungart et al. 2001; Freyman et al. 2001; Best et al. 2004; Culling et al. 2004; Freyman et al. 2004; Hawley et al. 2004; Brungart 2005; Edmonds and Culling 2005; Kidd et al. 2005; Shinn-Cunningham 2005; Rakerd et al. 2006; Best et al. 2006; Ihlefeld and Shinn-Cunningham 2008b; Ihlefeld and Shinn-Cunningham 2008a; McDermott 2009; Ruggles and Shinn-Cunningham 2011; Middlebrooks and Onsan 2012; Bremen and Middlebrooks 2013; Shinn-Cunningham et al. 2017; Leibold et al. 2019). Understanding the functional principles and neural correlates of the binaural listening advantage is not only valuable for basic auditory research, but could also aid the development of clinical assessment and intervention methods. Despite this, the majority of past scene analysis studies have focused on monaurally driven grouping cues or have employed stimuli, in which monaural and binaural grouping cues are present simultaneously, making it difficult to isolate the contribution of binaural cues on the results. In order to reliably assess the contribution of the binaural listening advantage to the perceptual organization of complex auditory scenes in neuroscientific experiments, a novel stimulation paradigm specific to binaural hearing would be highly beneficial.

In all perceptual experiments, a trade-off has to be made between the degree of control available over stimulus parameters and the ecological validity of the stimulation. Simple stimuli (e.g., noise or tone bursts) enable precise control over a limited set of stimulus parameters and a large number of identical trials to be run within a short experiment. Therefore, they are well suited for the trial-averaging-based analysis procedures employed in many neuroscientific experiments (Picton 2010; Schnupp et al. 2011), but bear little resemblance to the spectro-temporal complexity of real soundscapes. Consequently, experimental results obtained with simplistic stimuli may generalize poorly to explain auditory processing of complex natural scenes. The use of realistic stimuli (e.g., speech) is methodologically challenging, as precise manipulation of their low-level parameters may be difficult. Moreover, due to the variability inherent to natural sounds, realistic stimuli may not be ideally suited for analysis paradigms that capitalize on coherent neural activation across experimental trials. These limitations combined with the requirement for a large number of stimulus repetitions in EEG- and MEG-based measurements of auditory processing have led to the prevalent use of simplistic stimuli over ecologically valid, but methodologically challenging stimuli.

In recent years, efforts have been made towards developing novel synthetic stimuli for neuroscientific scene analysis studies that strike a balance between experimental control and ecological validity (see Discussion). Unfortunately, all of these stimuli introduce monaural grouping cues, and no spectro-temporally complex stimulation paradigm specific to binaural hearing has yet been established for use in neuroscientific experiments. Here, we propose that random-chord stereograms (RCS) could potentially provide such a paradigm.

RCS stimuli are a novel, binaurally driven class of auditory stimuli developed by Nassiri and Escabí (2008) that exploit time–frequency-specific interaural envelope correlation to induce salient, binaurally derived auditory percepts. They are similar to dichotic pitch stimuli driven by interaural phase disparity (IPD) (e.g., Cramer and Huggins 1958; Culling et al. 1998; Dougherty et al. 1998; Culling 1999; Johnson et al. 2003; Hautus and Johnson 2005), but can leverage a wider range of frequencies than what is possible with purely IPD-driven stimulation. RCS stimuli can be conceptualized as the auditory analog of the random-dot stereograms (RDS) (Julesz 1960; Julesz 1971) commonly used in stereopsis-based studies of binocular vision. Whereas RDS stimuli consist of a pair of noise-like images that appear random when viewed monocularly, but induce a visual “pop-out” effect when viewed under the appropriate binocular conditions, RCS stimuli are essentially noise under monaural listening, but yield an auditory reorganization effect under binaural listening. The perceptual details of this reorganization are determined by the spectro-temporal dynamics of the interaural envelope correlation manipulations.

Due to their flexibility, RCS stimuli show great promise as a stimulation paradigm for binaural scene analysis experiments. However, the suitability of these stimuli for neuroscientific studies has not yet been assessed. Here, we recorded EEG responses to RCS stimuli from 12 normal-hearing subjects. We wanted to inspect whether or not the temporal dynamics of RCS stimuli were reflected in the time- and frequency-domain representations of the event-related potentials (ERP) evoked by the stimuli. In addition, we used time–frequency decomposition to assess the frequency-specific phase coherence of the EEG responses measured across stimulus repetitions.

## Methods

### Subjects

12 staff members (3 females, mean age: 32 years, SD: $\pm 6.7$ years) from Aalto Acoustics Lab participated in the recordings. The participation was on a voluntary basis and the subjects received no compensation for participation. The experimental setup and procedures were approved by the Ethics Review Board of Aalto University. All participants gave a written form of informed consent before participating in the recordings.

**Fig. 1:**
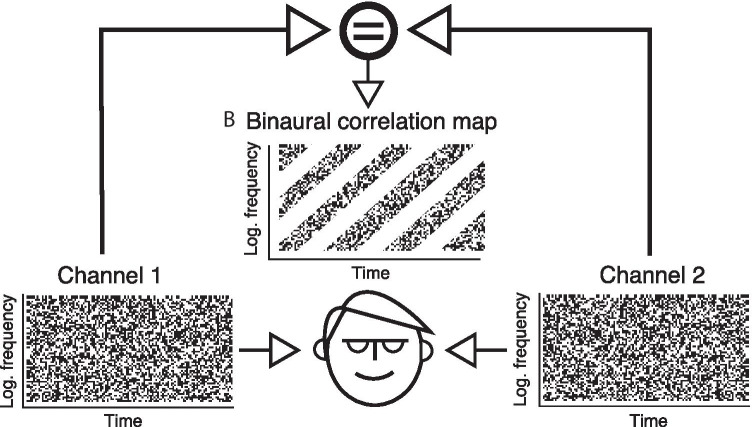
Stimulation paradigm for RCS stimuli. Each ear is presented with a noise-like sound with spectro-temporal patterns encoded into the envelope correlation of the left and right channels. The embedded patterns are perceivable only in binaural listening

**Fig. 2:**
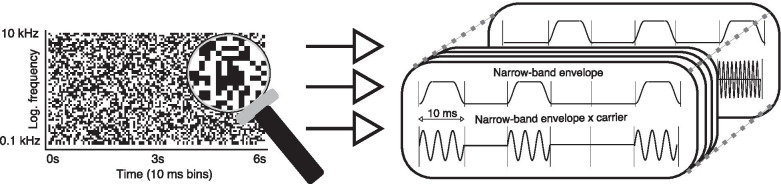
Schematic illustration of the structure of a single RCS stimulus channel. A random time–frequency-domain binary map determines which frequency bands are gated at each 10-ms time bin. White and black matrix pixels denote time–frequency elements where the corresponding narrow-band envelope has a value of one or zero, respectively. Envelopes are generated and applied independently to each sinusoidal frequency in the tone cluster

**Fig. 3:**
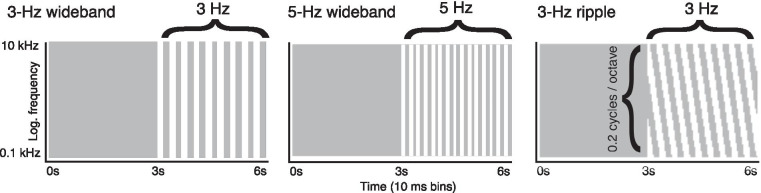
Correlation matrices for the RCS stimuli used in the EEG recordings. Gray pixels denote time–frequency elements that are gated independently in the left and right channels. Conversely, white pixels denote time–frequency elements that are gated coherently across the left and right channels. All three stimuli consisted of an initial 3-s unmodulated segment and a subsequent 3-s segment with RCS modulations. For wideband stimuli, RCS modulations were applied to all time–frequency elements at rates of 3 or 5 Hz. In the ripple stimulus, the correlation matrix consisted of shifting regions of correlated and uncorrelated frequency bands resulting in a spectro-temporal ripple pattern in the binaural envelope correlation

### Stimuli

RCS stimuli exploit interaural envelope correlation to drive perceptual reorganization of binaurally presented noise. Conceptually, they are similar to the random-dot stereograms used widely in studies of binocular vision, in that they contain binaurally encoded stimulus features that emerge perceptually only under binaural listening (see Fig. 1). Here, the RCS synthesis procedure follows the steps outlined in Nassiri and Escabí (2008), but also deviates from that study in some minor details.

The stimuli were synthesized digitally in MATLAB (Mathworks, Natick MA) at a 48-kHz sample rate. First, a noise-like cluster of *k* random-phase carrier sinusoids *x*(*k*, *t*) was synthesized according to:

1 $$\begin{aligned} x(k,t) = \sin (2 \pi f_k t + \phi _k), \end{aligned}$$

where *k* is a frequency index variable, *t* is the time index running up to 6 seconds, and $\phi _k$ is the frequency-specific starting phase of the carrier, assigned randomly from the range $[0, 2\pi ]$. The cluster was used as the temporal fine structure of both the left and right channels of the stimuli. The carrier frequencies were chosen according to:

2 $$\begin{aligned} f_k = f_1 \cdot 2^{k \cdot \Delta X}, \end{aligned}$$

where $f_1 = 100$ Hz and $\Delta X$ determines the separation of the cluster components in logarithmic frequency space. Here, as in Nassiri and Escabí (2008) a $\Delta X$ of 0.038 was used, but the frequency index *k* ran from 0 to 175, resulting in 176 frequency components spanning the range from 100 Hz to approximately 10 kHz. This range of partials was chosen to keep the carrier frequencies within the flat pass band of the earphones used to deliver the stimuli (see Section Stimulus Presentation).

Next, a 176-by-600 random binary matrix (with the rows corresponding to the *k* frequency indices and the columns to 600, 10-ms temporal segments in the tone cluster) was used to determine the stochastic gating (envelopes) of the individual time–frequency elements in one channel of the stimulus. The binary matrix for gating the other channel was then created in a similar manner, with the exception that a time–frequency correlation matrix, $\rho _{k,l}$, described by

3 $$\begin{aligned} \rho _{k,l} = \mathrm{round} [0.5 + 0.5 \sin (2 \pi (\Omega X_k + F_m T_l))], \end{aligned}$$

was used to control the envelope correlation of specific time–frequency elements between the left and right channels during the latter half (i.e., 3 - 6 s) of the stimulus. Here, the notation again follows that of Nassiri and Escabí (2008); namely, $X_k = \log _2 (f_k / f_1)$ is the frequency axis, $T_l = l \cdot T$ is the time axis discretized to 10-ms steps, $\Omega$ is the ripple density expressed in cycles per octave and $F_m$ is the RCS modulation frequency. A correlation matrix value of 1 denotes that the gating of the corresponding time–frequency element is correlated across the left and right channels (i.e., the time–frequency element corresponding to the correlation matrix element is gated identically in both channels). Conversely, correlation matrix values of 0 denote that the gating of the corresponding time–frequency element is determined randomly (50% probability per gating condition) for both channels. The gating matrix for the latter half of the stimuli (3 - 6 s) of the left channel was then formed according to the correlation matrix. Finally, a binary-like envelope was imposed on each time–frequency element of both channels according to the values in the respective gating matrices. The gating was implemented in 10-ms segments using raised-cosine ramps with 0.5-ms rise and fall times (see Fig. 2).

We chose three RCS stimuli for the EEG recordings, namely two stimuli where $\rho$ was modulated between 0 and 1 across all carrier frequencies at rates of 3 or 5 Hz (i.e., $\Omega = 0$, $F_m =$ 3 or 5 in Eq. (3)). In addition, we chose a stimulus where $\rho$ was modulated in shifting frequency bands according to a 3-Hz spectro-temporal ripple with a ripple density of 0.2 cycles per octave ($\Omega = 0.2$, $F_m = 3$). For the remainder of the text, we refer to these stimuli as “3-Hz wideband,” “5-Hz wideband” and “3-Hz ripple” according to the rate ($F_m$) and type of modulation ($\Omega$) used in the stimulus synthesis. The correlation matrices for the chosen stimuli are shown schematically in Fig. 3.

The initial 3-s segments of all three stimuli were qualitatively the same as they contained no RCS modulations; the percepts evoked by the initial segments can be described as two auditory images of wideband noise lateralized to the two ears. When RCS modulations were introduced after 3 s from stimulus onset, the wideband modulations resulted in a rapid perceptual shift from two lateralized images to a single fused image at the auditory midline. During the ongoing RCS modulations, percepts cycled between two lateralized images and a single fused image. In the case of the ripple stimulus, the introduction of the RCS modulations did not result in a single fused image, rather, two lateralized noise images remained at the two ears and a percept of a spectro-temporal ripple appeared. Further details on the perceptual aspects of RCS stimuli obtained from behavioral studies are documented in the original RCS manuscript by Nassiri and Escabí (2008).

### Stimulus Presentation

For the EEG recordings, a set of 10 independently generated samples was prepared for each of the three stimulus types, out of which the test software randomly selected one for presentation in each trial. The samples had the same correlation matrix shape and fine structure parameters, but differed in the stochastic aspects of the stimuli, i.e., the initial starting phase $\phi _k$ of the carrier sinusoids and the binary gating matrices of both the left and right channels were randomized independently for each of the 10 samples. As such, the samples were qualitatively the same, but differed in the stochastics of the time–frequency-domain elements. This procedure was applied to introduce variation to the spectro-temporal details of the stimuli while simultaneously ensuring that the pattern of interaural envelope correlations remained constant across trials.

Stimuli were presented over ER-2 Tubephone (Etymotic Research, Elk Grove Village, IL, USA) insert earphones through an RME Fireface UCX (RME Audio, Germany) sound card, at a sample rate of 48 kHz. All stimuli were presented at a monaural sound level of 68 dBA.

### EEG Data Acquisition

**Fig. 4:**
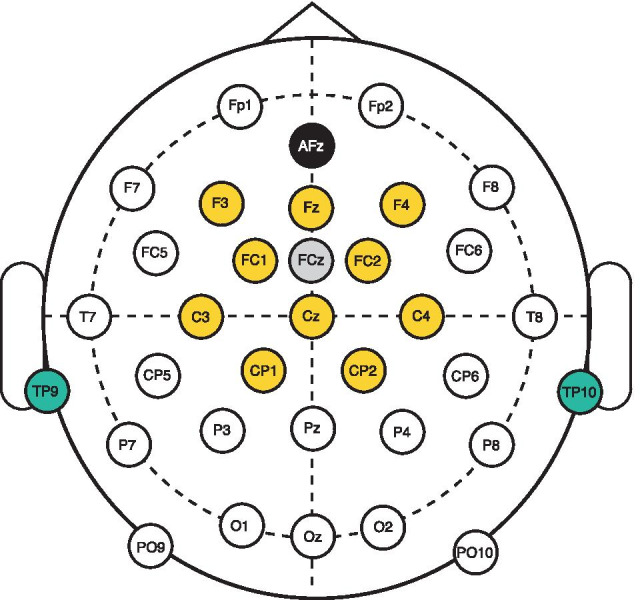
Topographical view of the electrode montage used in the recordings. During the recordings, FCz (gray) was used as the reference electrode and AFz (black) as the ground electrode. The yellow electrodes denote the fronto-central cluster chosen for data analysis. The teal-colored electrodes were used to form the pseudo-mastoid reference used in offline processing

EEG responses were measured in an electrically shielded and sound-proofed room at the Aalto Behavioral Laboratory at Aalto University. A 32-channel active electrode array fitted on an actiCAP (BrainProducts GmbH, Munich, Germany) was used to measure the scalp potentials; the electrode array was powered via a PowerPack (BrainProducts GmbH, Munich, Germany) power supply. A schematic view of the electrode montage is shown in Fig. 4. The arrangement of the electrodes followed the ten-twenty system (Klem et al. 1999). Electrodes FCz and AFz were used as the respective reference and ground electrodes during the recordings. Signals from the electrode array were routed to a BrainAmp (BrainProducts GmbH, Munich, Germany) amplifier and digitized at a sample rate of 500 Hz. The signals were then stored on a desktop computer running BrainVision Recorder (BrainProducts GmbH, Munich, Germany).

Since the EEG recordings did not involve a psychophysical task that the subjects had to engage in, subjects were instructed to ignore the stimuli and concentrate on reading a text of their own choosing during the measurement sessions. This was done to facilitate the comfort of the subjects. The recordings were segmented into six 10 - 15 min blocks (two blocks per each of the three stimuli). The order of the blocks was randomized for each subject, with the constraint that the same stimulus was never repeated in back-to-back blocks. The subjects were free to take breaks from the recordings between blocks. The inter-stimulus interval was 3 s in all blocks. The experiment was sequenced using Presentation software (Neurobehavioral Systems, Inc., Berkeley, CA).

### EEG Data Preprocessing

The EEGLAB MATLAB toolbox (Delorme and Makeig 2004) was used for processing the measurement results. The offline data preprocessing pipeline consisted of the following steps: re-referencing the data from all electrodes to a pseudo-mastoid reference using the mean of electrodes TP9 and TP10 (see Fig.4), downsampling the data to a 100-Hz sample rate and band-pass filtering the data between 1 and 40 Hz. The filtering was implemented using a 331-point finite impulse response filter with cutoff frequencies (-6 dB) at 0.5 and 40.5 Hz; the width of the transition bands was 1 Hz. The magnitude response of the filter outside the transition band edges was below -50 dB. Zero-phase filtering was used to preserve the phase spectrum (de Cheveigné and Nelken 2019).

Data were epoched into segments encompassing 1500 ms before and 7500 ms after stimulus onset, yielding epochs with a 1.5-s silent period on either side of the 6-s stimulus segments. Each epoch was then normalized according to a 250-ms pre-stimulus baseline average.

After epoching, the data from each subject were inspected visually for epochs contaminated with excessive noise due to movements, biting and other high-magnitude artefacts. Contaminated epochs were rejected manually. At least 113 epochs remained for all subjects and conditions after epoch rejection. The total number of retained epochs across subjects were 1610, 1627, 1552 for the 3-Hz wideband, 5-Hz wideband and 3-Hz ripple stimuli, respectively.

The remaining epochs were then processed with the infomax independent components analysis algorithm provided by the EEGLAB toolbox (Delorme and Makeig 2004). The resulting independent components were inspected visually at the levels of activation time courses, frequency-domain characteristics and topographical distribution. In an effort to avoid losing stimulus related activity, data rejection at the component level was conservative and only the components identified as blinks and saccade artefacts (as indexed by their characteristic topographical distributions and activation times that appear unrelated to the stimulation time course) as well as electrode contact noise (characterized by stochastic, high-frequency activations, sharply localized to single electrodes) were removed from the data sets (Cohen 2014; Hari and Puce 2017).

### ERP Analyses

An initial inspection of the topographical arrangement of the grand-average electrode ERPs revealed that the electrodes in the fronto-central cluster consisting of the electrodes: F3, Fz, F4, Fc1, Fc2, C3, Cz, C4, Cp1, Cp2 (yellow electrodes in Fig. 4, referred to as the “fronto-central electrodes” for the remainder of the text) showed qualitatively similar responses. Therefore, the grand-average ERPs were computed as the average of the responses in the fronto-central electrodes and the 12 subjects; ERP variability was quantified by the standard error measured across subjects. In addition, we extracted subject-specific peak-to-peak magnitudes for the cN1–cP2 complex occurring after the introduction of the RCS modulations and subjected these data to similar nonparametric statistical procedures as our other data to discover whether the peak-to-peak magnitudes varied with the type of RCS modulation applied (see Secs. Frequency-Domain Analyses and ITC Analyses for details on the statistical procedures employed throughout the analyses).

### Frequency-Domain Analyses

In the frequency domain, our main interest was to assess the degree of spectral power increase in the EEG responses at the fundamental (F0) and first two harmonic frequencies (F1, F2) of the RCS modulations during the latter segments (3-6 s) of the stimulation relative to the initial (0-3 s), unmodulated segments. To that end, the steady-state responses of the initial segments (1 - 3 s after stimulus onset, referred to as “segment 1”) and latter, modulated segments (4 - 6 s after stimulus onset, referred to as “segment 2”) of the ERPs averaged across the fronto-central cluster were extracted into Hann-windowed vectors and processed with a Fourier transform. Here, we were specifically interested in the steady-state responses and therefore excluded the transient segments (onset response: 0-1 s, change onset response: 3-4 s and offset response: 6 s onward) from the frequency-domain analyses.

The statistical significance of the spectral power increase was assessed with nonparametric statistical tests between the power spectral density (PSD) measures at F0, F1 and F2, obtained from segments 1 and 2, for each of the three stimulus types. Friedman tests were used for omnibus testing the group-level PSD differences for each stimulus. The Friedman test is a nonparametric test suitable for repeated-measures data that deviates from the assumptions of normality and homogenous variances between the test samples that parametric tests rely on. This procedure was preferred over the more common analysis of variance due to the fact that the variances in the PSD measures were non-homogenous between sample groups. For example, PSD variance at F0 and F1 was much higher between subjects in segment 2 than in segment 1. For data that yielded a statistically significant results for the Friedman tests, pair-wise comparisons were carried out between the PSD measures at the F0, F1 and F2 obtained from the two segments, using the exact version of one-directional Wilcoxon signed-rank tests (e.g., F0 PSD in segment 2 > F0 PSD in segment 1). One-directional tests were used since a frequency-specific *increase* in PSD was expected in segment 2 relative to segment 1. Similar to the Friedman test, the Wilcoxon test is a nonparametric analogue to the paired-samples t-test, suitable for non-normal paired data.

### ITC Analyses

In the time–frequency domain, our main interest was to supplement the frequency-domain power-increase analyses with phase-angle time-series data. To this end, we evaluated the inter-trial phase coherence (ITC) (Tallon-Baudry et al. 1996; Delorme and Makeig 2004) to see how systematically the obtained responses follow the RCS modulations across repetitions of the same stimuli. ITC analyses exploit the EEG phase-angle time series obtained via time–frequency decomposition of the electrode signals and quantify the phase coherence for each time–frequency bin as the length of the normalized phasor at that time–frequency bin, computed across all trials for each channel of EEG. As such, ITC values are constrained between values of 0 and 1, with 1 corresponding to perfect phase-angle alignment across all trials, and ITC of 0 to randomly distributed or perfectly antiphasic phase angles across all trials (Cohen 2014). Here, ITC is used to supplement the power spectrum analyses by assessing the trial-to-trial consistency of the EEG responses.

The time–frequency decomposition was implemented with a wavelet transform across the frequency range 2 - 20 Hz in 0.25-Hz increments. This range was chosen over the entire frequency range of the downsampled EEG signals, since an initial analysis encompassing also the upper frequency range did not reveal any additional effects and the response details at the relatively low frequencies of the RCS modulations are better visualized with the limited frequency span. The wavelet kernels contained 3 cycles at the lowest frequency and expanded to 15 cycles at 20 Hz. The transforms were evaluated at 400 time points at each frequency. The ITC decompositions were computed for all electrodes in the fronto-central cluster and each subject separately. For visualization purposes, ITC values were averaged across subjects and fronto-central electrodes and presented as a grand average for each of the three stimulus types.

For statistical testing of the results, we used the mean ITC values at F0 and F1 of the RCS modulations across the temporal segments 1-2 s and 4-5 s (referred to as segments 1 and 2, respectively), averaged across the electrodes in the fronto-central cluster. The second harmonic (F2) was excluded from the statistical evaluation of the ITC results since no effects specific to F2 were revealed in the ITC topographies nor the frequency-domain analyses. This collapsed the high-dimensional ITC data into four ITC measures per stimulus condition for each subject, namely mean ITC in the steady-state responses prior to the onset of the RCS modulations (segment 1) at F0 and F1, and the corresponding measures in the responses during the RCS modulation (segment 2).

The onset (0-1 s), change onset (3-4 s) and offset (6 s onward) responses were excluded from the group-level statistical analysis, as we wanted to assess the ITC differences between the modulated and unmodulated steady-state responses. Including the onset, change onset and offset responses in this analysis would have biased the ITC estimates due to the relatively high inter-trial consistency of these transient responses. Similar to the frequency-domain analyses, we expected the introduction of the RCS modulations in segment 2 to *increase* the ITC at F0 and F1 relative to segment 1. Accordingly, the statistical significance of the difference in the segment-wise ITCs for each stimulus was assessed using the exact version of the one-sided Wilcoxon tests (e.g., ITC at F0 in segment 2 > ITC at F0 in segment 1).

To supplement the group-level analyses, we also explored the statistical significance of ITC across the entire stimulation time course at the subject level. For these analyses, we assessed whether the distribution of phase angles obtained via the wavelet transforms deviated from a von Mises distribution (i.e., the circular equivalent of a normal distribution, Stephens 1969) to a statistically significant degree, using the threshold value for ITC given by the equation

4 $$\begin{aligned} ITC _{Threshold } = \sqrt{\frac{- \mathrm{log} (\alpha )}{N}}. \end{aligned}$$

Here, $\alpha$ is the p-value threshold for statistical significance ($\alpha = .05$ in all our analyses) and *N* is the subject- and stimulus-specific number of trials used for computing the ITC values (Cohen 2014). These analyses provide additional insight into the phase consistency of the EEG temporal dynamics and highlight intersubject differences that are obscured by group-level summary results.

## Results

**Fig. 5:**
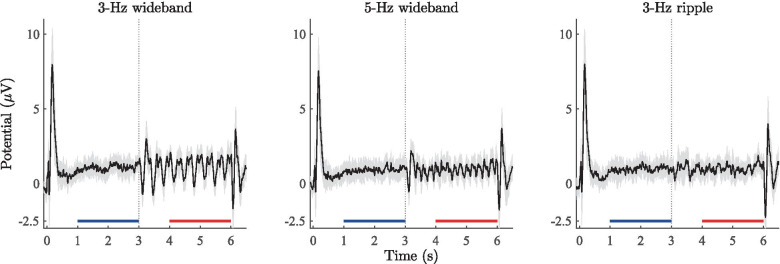
Grand-average ERPs (1610, 1627 and 1552 repetitions) from 12 subjects measured across the electrodes in the fronto-central cluster (see Fig.4). Shaded regions denote the standard deviation of the ERPs across subjects. The vertical line at 3 s denotes the onset of the RCS modulations. The blue and red horizontal lines denote the time periods (segments 1 and 2) of the ERPs extracted for spectral analysis (see Sec. Frequency-Domain Results for details)

The grand-average ERPs are shown in Fig. 5. Qualitatively, the responses consist of five distinct regions: 1) the large deflection at the stimulus onset (0 - 1 s), 2) the steady-state response during the noise segment where the interaural envelope correlation is zero (segment 1: 1 - 3 s), 3) a change-onset response around 3 s, where the RCS modulations begin, 4) the steady-state response during the RCS modulations from 4 s onward (segment 2: 4 - 6 s) and 5) the offset response at 6 s.

In the case of the wideband stimuli, the introduction of the RCS modulations 3 s after stimulus onset evoked a visible steady-state response in the ERPs that appears to follow the RCS modulations. The magnitude of the 3-Hz steady-state response is larger than in the case of the 5-Hz stimulus. No steady-state response is visible in the case of the 3-Hz ripple stimulus. Overall, the grand-average ERPs show that the time course of wideband RCS modulations is reflected in the time-domain representation of the ERPs. A cross-correlation computation between the 3-Hz wideband RCS modulation pattern and the associated steady-state ERP yielded the highest correlation value at a lag time of 110 ms. This latency suggests that the observed steady-state responses originate from cortical, rather than subcortical neural substrates.

**Fig. 6:**
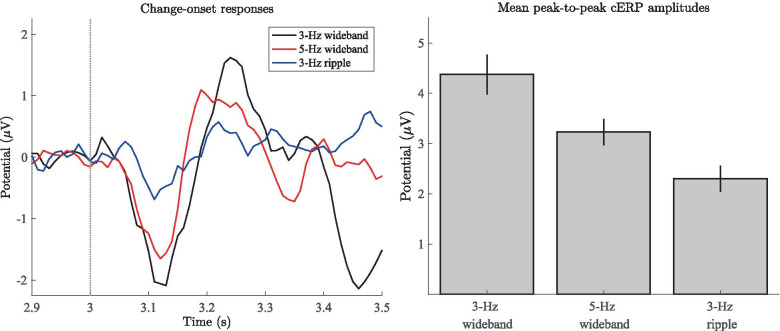
Left panel: Grand-average change-onset responses. Data as in Fig. 5, but baseline corrected according to a 100-ms time period prior to the onset of the RCS modulations (i.e., 2.9 - 3.0 s). Right panel: Mean peak-to-peak amplitudes of the change-onset responses. Error bars denote the standard error across 12 subjects

The mean change-onset responses evoked by the introduction of the RCS modulations are shown in the left-hand side panel of Fig. 6 for each stimulus type. The latencies of the cN1 peaks are comparable to those reported in the literature for other stimulus types (Halliday and Callaway 1978; Ungan et al. 1989; McEvoy et al. 1990; McEvoy et al. 1991; Sams et al. 1993; Jones et al. 1991; Chait et al. 2005; Dajani and Picton 2006), i.e., 110-130 ms after the onset of the modulations. This corresponds to an additional latency of 30 - 50 ms relative to the N1 deflection in the sound onset complex, indicating that changes in binaural envelope correlation are processed with similar cortical latencies as in the case of other previously reported binaural parameters.

The average peak-to-peak amplitudes for the cN1–cP2 complex of each stimulus type are shown in the right-hand side panel of Fig. 6. Qualitatively, it is apparent that the peak-to-peak magnitudes are highest for the two wideband stimuli and significantly lower for the ripple stimulus. Statistical assessment of the peak-to-peak magnitudes using the Friedman test revealed a statistically significant ($\alpha = .05$) group-level difference between the three stimulus types ($\chi ^2 (2) = 15.17, p \le .001$). Pair-wise comparisons using one-sided Wilcoxon tests confirmed that the peak-to-peak magnitudes were significantly different between all three stimulus types, with both of the wideband stimuli yielding larger response amplitudes than the ripple stimulus (see Table 1).

**Table 1:** Wilcoxon comparisons of the peak-to-peak amplitudes of the change-onset responses obtained for the three stimuli. All p-values are Bonferroni corrected. Bold p-values denote statistically significant results ($\alpha = .05$)

**Change-onset response amplitude**	**W(12)**	**p**
3-Hz wideband > 5-Hz wideband	7	**.014**
3-Hz wideband > 3-Hz ripple	0	$\le$ **.001**
5-Hz wideband > 3-Hz ripple	10	**.032**

### Frequency-Domain Results

**Fig. 7:**
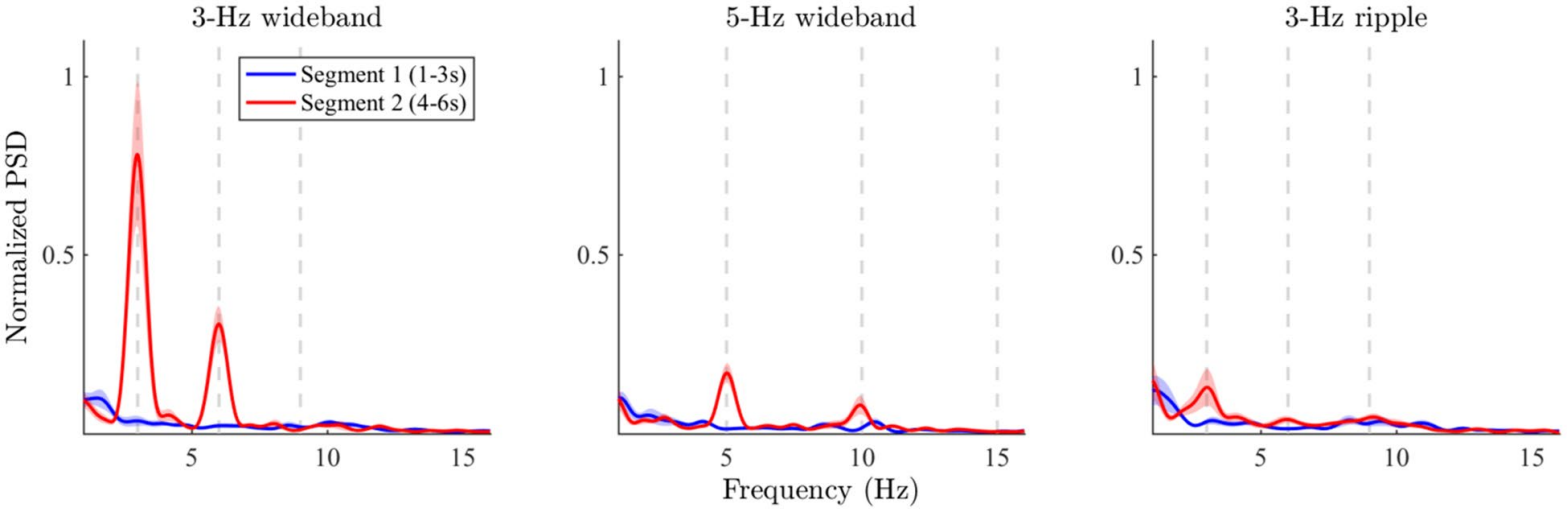
Average power spectra measured across the fronto-central electrodes for unmodulated steady-state stimulus segments (segment 1, 1-3 s post stimulus onset, blue traces) and RCS-modulated steady-state segments (segment 2, 4 - 6 s post stimulus onset, red traces). Amplitudes are normalized to a common peak value. The shaded regions denote the standard error measured across the 12 subjects. The vertical gray lines denote the fundamental frequency (F0) and the two lowest harmonics (F1 and F2) of the RCS modulation frequency for each stimulus. The 3-Hz wideband stimulus evoked the largest activity at F0 and F1 of the RCS modulations (i.e., 3 and 6 Hz). The spectral power at the corresponding harmonics for the 5-Hz stimulus was lower than in the 3-Hz case, but higher than in the unmodulated segments. The lowest amplitudes were measured for the 3-Hz ripple stimulus

The spectral analysis results are shown in Fig. 7 for segments 1 (blue trace) and 2 (red trace). Qualitative inspection of the results from the 3-Hz wideband stimulus (leftmost panel in Fig. 7) shows a prominent increase in PSD at the fundamental frequency (3 Hz) and first harmonic (6 Hz) of the RCS modulation frequency during the modulated segment (red trace), but the PSD estimates are similar between the two segments at all other frequencies. The results from the 5-Hz wideband stimulus (middle panel in Fig. 7) are qualitatively similar to those from the 3-Hz stimulus, but the PSD differences between the segments are much less prominent in the case of the 5-Hz stimulus. The results for the 3-Hz ripple stimulus (rightmost panel in Fig. 7) show the weakest PSD increase at the corresponding frequencies.

Friedman tests were statistically significant ($\alpha = .05$) across the six PSD values in each of the three stimulus conditions (3-Hz wideband: $\chi ^2 (5) = 43.76, p \le .001$, 5-Hz wideband: $\chi ^2 (5) = 37.67, p \le .001$, 3-Hz ripple: $\chi ^2 (5) = 11.76, p = .038$), indicating that statistically significant differences exists between the PSD values obtained from the two temporal segments (prior and after the onset of RCS modulations) and the three frequencies of interest (F0, F1 and F2) for all of the three stimuli. Results of further statistical assessments with pair-wise Wilcoxon tests are shown in Table 2. In summary, the statistical assessment of the frequency-domain results shows that the introduction of the RCS modulations yielded a significant PSD increase at the fundamental frequency and the first harmonic for both of the wideband stimuli, while similar increases for the ripple stimulus were much less prominent and failed to remain statistically significant after p-value correction.

**Table 2:** Wilcoxon comparisons of PSD at the RCS modulation frequency (F0) and its first two harmonics (F1, F2) during the modulated (segment 2) and unmodulated (segment 1) segments of the stimulation. p-values presented as in Table 1. “$\dagger$” denotes a p-value that was statistically significant before Bonferroni correction

**Segment 2 PSD > segment 1 PSD**	**W(12)**	**p**
3-Hz wideband F0	0	$\le$ **.001**
3-Hz wideband F1	0	$\le$ **.001**
3-Hz wideband F2	46	>.05
5-Hz wideband F0	0	$\le$ **.001**
5-Hz wideband F1	1	**.002**
5-Hz wideband F2	46	>.05
3-Hz ripple F0	15	>$.05^{\dagger }$
3-Hz ripple F1	17	>$.05^{\dagger }$
3-Hz ripple F2	21	>.05

**Fig. 8:**
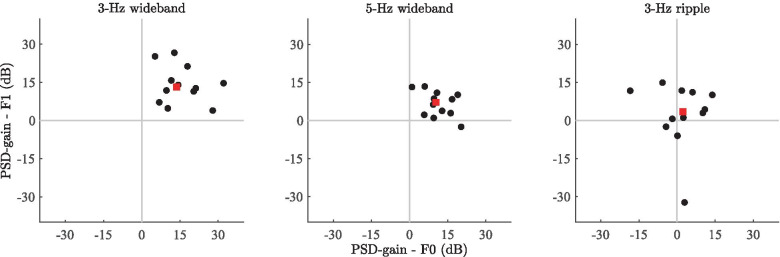
Scatter plots of the subject-wise gains in power spectral density between segment 1 (1-3 s) and segment 2 (4-6 s) of the subject ERPs. F0 and F1 denote the fundamental frequency and first harmonic of the RCS modulations, respectively. Data points correspond to the PSD measurements from individual subjects. The red square corresponds to the median F0 and F1 coordinates of the data points. In the case of the 3-Hz wideband stimulus, all points are within the upper-right quadrant of the plot, indicating that PSD was higher for all subjects in segment 2 than in segment 1 for both F0 and F1; The magnitude of the F0 and F1 gains vary across subjects. Similarly, for the 5-Hz wideband stimulus, most data points lie in the upper-right quadrant, indicating a PSD increase at both frequencies for most subjects. Further, the magnitudes for the 5-Hz stimulus are lower (data points closer to origin) than in the case of the 3-Hz stimulus. For the 3-Hz ripple stimulus, the points are more widely spread across the quadrants, and the cluster median (red square) is near the origin, suggesting no systematic effects

Figure 8 shows the scatter plot of the subject- and frequency-specific PSD gains (i.e., 10log$_{10}$(PSD segment 2 / PSD segment 1)) across the two steady-state segments. The x- and y-axes denote the dB difference in PSD measured at F0 and F1 of the RCS modulations, respectively; positive values denote an increase in PSD during RCS modulation relative to the preceding, unmodulated steady-state segment. Here, the general trends verified by the statistical analyses are supplemented by displaying the variability between individual subjects. For the 3-Hz wideband stimulus, the data point cluster is in the upper-right quadrant, indicating a PSD increase across subjects for both F0 and F1. The data from the 5-Hz wideband stimulus show a similar, albeit less prominent effect, with the cluster centroid (denoted by the red square) closer to the origin than in the case of the 3-Hz stimulus. Whereas with the 3-Hz stimulus, inter-subject variability is apparent—as shown by the relatively large spread of the individual data points—the amount of inter-subject variability is smaller for the 5-Hz stimulus, as indicated by the fact that the data points are relatively tightly clustered at smaller values along both axes. In line with the statistical analyses, the scatter plot for the 3-Hz ripple stimulus is centered close to the origin, implying a lack of a significant effect along either axis.

Overall, the frequency-domain analyses show that the RCS modulations increased the power spectral density of the steady-state EEG responses at the fundamental frequency and the first harmonic of the modulation frequency. While the largest spectral power increase was observed with the 3-Hz wideband stimulus, a qualitatively similar effect was observed with the 5-Hz wideband stimulus, albeit at a lower magnitude. The 3-Hz ripple stimulus on the other hand showed no statistically significant PSD increase in the steady-state response at the corresponding frequencies, indicating that the magnitudes of the responses depend not only on the modulation frequency, but also on the type of RCS modulation.

### ITC Results

**Fig. 9:**
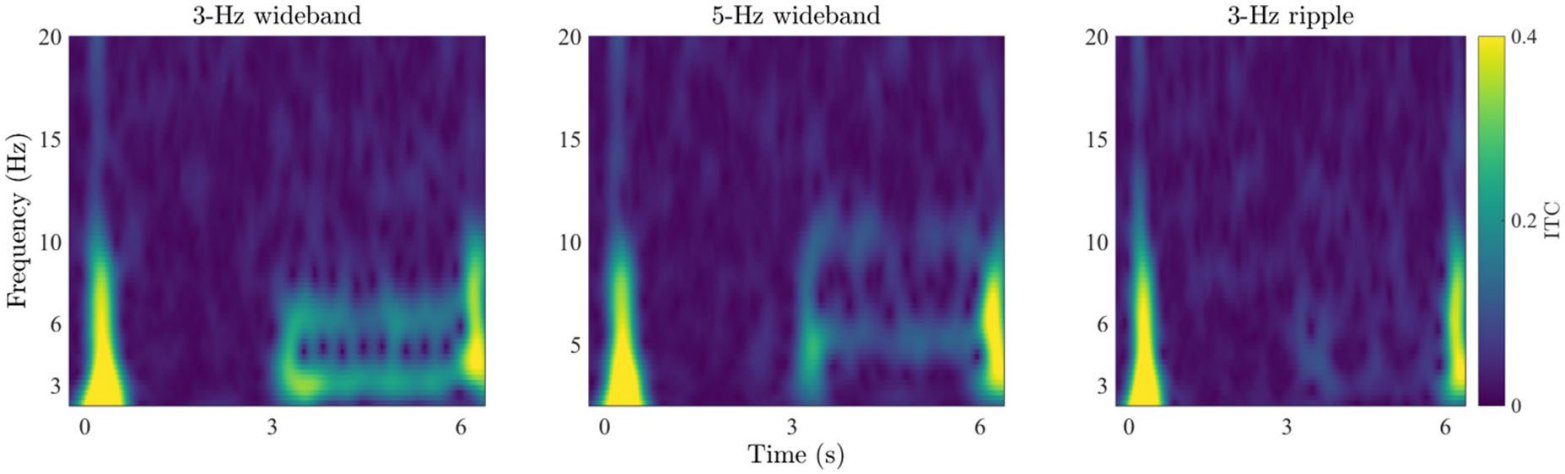
Mean ITC responses computed across the fronto-central electrode cluster for the three stimuli. The color scale clips at 0.4

The grand-average ITC plots for the two wideband stimuli are shown on the left and middle panels of Fig. 9. Here, the major features in the ERPs are visible in the time–frequency-domain phase coherence. The onset (0-1 s) and offset responses (6 s) display a high degree of inter-trial coherence, indicating—as expected—consistent responses to these segments in the stimulation time course. The introduction of the RCS modulations at 3 s is visible as increased phase coherence at F0 (3 or 5 Hz) as well as F1 (6 or 10 Hz), indicating that the spectral power increases at the modulation frequencies were also phase consistent across trials. As in the case of the frequency-domain results, also the ITC measures are higher for the 3-Hz stimulus than for the 5-Hz stimulus, indicating that the decrease in spectral power with increasing modulation frequency observed here and in previous studies (e.g., Dajani and Picton 2006) is accompanied with a decrease in ITC. Further, the RCS modulations yielded no visible ITC increase at F2 of either stimulus nor at any other frequency unrelated to the modulation rate, indicating that the features visible in the time-domain ERPs are specific to the fundamental and first harmonic of the RCS modulation frequencies.

**Fig. 10:**
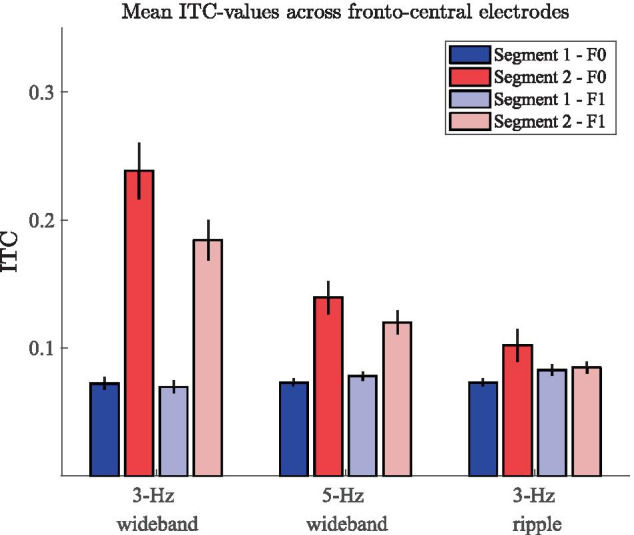
Mean ITC values at the fundamental frequency (F0) and first harmonic (F1) of the RCS patterns measured across the unmodulated segment 1 (here, 1-2 s, denoted by blue bars) and RCS modulated segment 2 (here, 4-5 s, denoted by red bars) of the three stimulus types. Error bars denote the standard error measured across 12 subjects

The right panel of Fig. 9 shows the corresponding ITC plots for the 3-Hz ripple stimulus. Here, only the onset and offset responses are visibly coherent across trials, suggesting that despite the perceptual saliency of the stimulation, the cortical responses were not evoked in a coherent manner across trials. Although there are minor signs of increased ITC at the modulation onset (3 s), ITC is not sustained during the ongoing RCS modulations as was observed in the steady-state responses with wideband modulations.

The average segment- and frequency-specific ITC values measured across subjects and electrodes extracted for the statistical analyses are shown in Fig. 10 for each stimulus. Friedman tests on the four ITC measures (mean ITC at F0 and F1 in segments 1 and 2) obtained for each stimulus revealed a statistically significant effect in the case of the 3-Hz wideband stimulus ($\chi ^2 (3) = 29.7, p \le .001$) and the 5-Hz wideband stimulus ($\chi ^2 (3) = 18.3, p \le .001$) but not for the 3-Hz ripple stimulus ($\chi ^2 (3) = 5.7, p = .13$), indicating that the RCS modulations resulted in an ITC increase during segment 2 relative to segment 1, only in the case of the two wideband stimuli. Results of further statistical assessments using pair-wise Wilcoxon tests are shown in Table 3. These analyses yielded a statistically significant result for both wideband stimuli at F0 as well as F1.

**Table 3:** Wilcoxon comparisons of ITC at the RCS modulation frequency (F0) and its first harmonic (F1) during the modulated (segment 2) and unmodulated (segment 1) segments of the stimulation. p-values presented as in Table 2. Ripple stimuli and ITC values at the second harmonic (F2) were not tested due to statistically insignificant p-values from previous analyses

**Segment 2 ITC > segment 1 ITC**	**W(12)**	**p**
3-Hz wideband F0	0	$\le$ **.001**
3-Hz wideband F1	0	$\le$ **.001**
5-Hz wideband F0	3	**.002**
5-Hz wideband F1	1	$\le$ **.001**

**Fig. 11:**
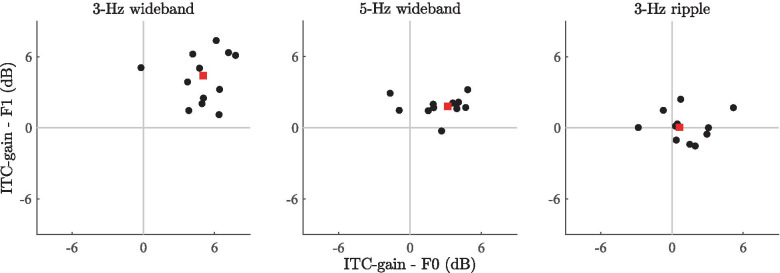
Scatter plots of the subject-wise changes in mean ITC between segment 1 (1-2 s) and segment 2 (4-5 s) of the subject responses. F0 and F1 denote the fundamental frequency and first harmonic, respectively, of the RCS modulations. Data points correspond to the mean ITC measurements from individual subjects. The red square corresponds to the median F0 and F1 coordinates of the data points. In the case of the 3-Hz wideband stimulus, most data points are in the upper-right quadrant of the plot, indicating that ITC increased systematically in segment 2 relative to segment 1. Similarly, for the 5-Hz wideband stimulus, most data points lie in the upper-right quadrant, indicating an increase in ITC at both frequencies for most subjects, but gains in ITC magnitudes are lower than in the case of the 3-Hz stimulus. For the 3-Hz ripple stimulus, the points are more widely spread across the quadrants and clustered near the origin, suggesting variable responses across subjects and a lack of general-level effects on ITC

Scatter plots of the subject-level ITC gains (i.e., 10log$_{10}$(ITC segment 2/ITC segment 1)) across segments 1 and 2 are shown in Fig. 11. Here, the overall trends are similar to the scatter plots for the PSD gains. For the 3-Hz wideband stimulus, the average ITC increased for all subjects during segment 2. (Data points are clustered in the upper-right quadrant, indicating an ITC increase at F0 and F1.) The results for the 5-Hz stimulus are similar, but reflect ITC gains of lower magnitude (cluster closer to origin) and less variability between subjects. In the case of the 3-Hz ripple stimulus, the cluster is near the origin and spread across the quadrants, suggesting no systematic ITC variations due to the ripple-shaped RCS modulations.

**Fig. 12:**
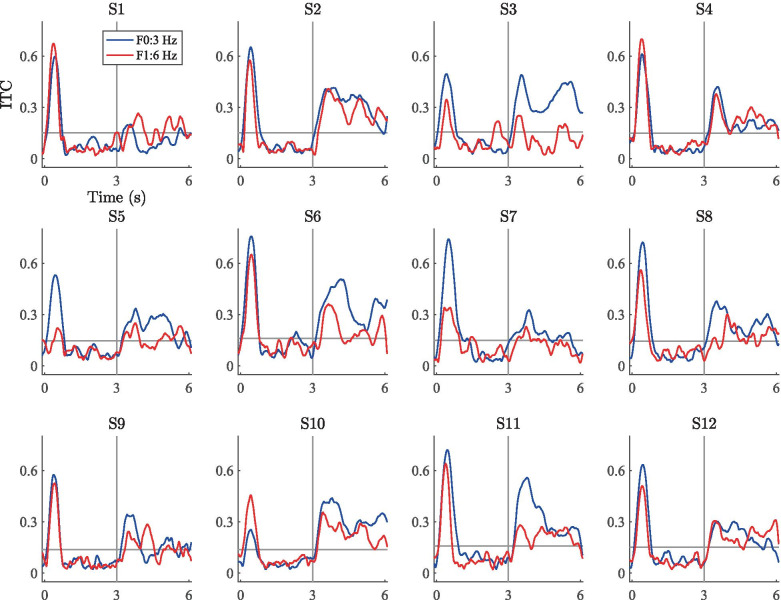
ITC time series for the 3-Hz wideband stimulus. The blue and red traces correspond to the fundamental frequency and the first harmonic, respectively. The horizontal gray line denotes the subject-specific threshold value for statistical significance according to Eq. (4). The vertical line denotes the onset of the RCS modulation

**Fig. 13:**
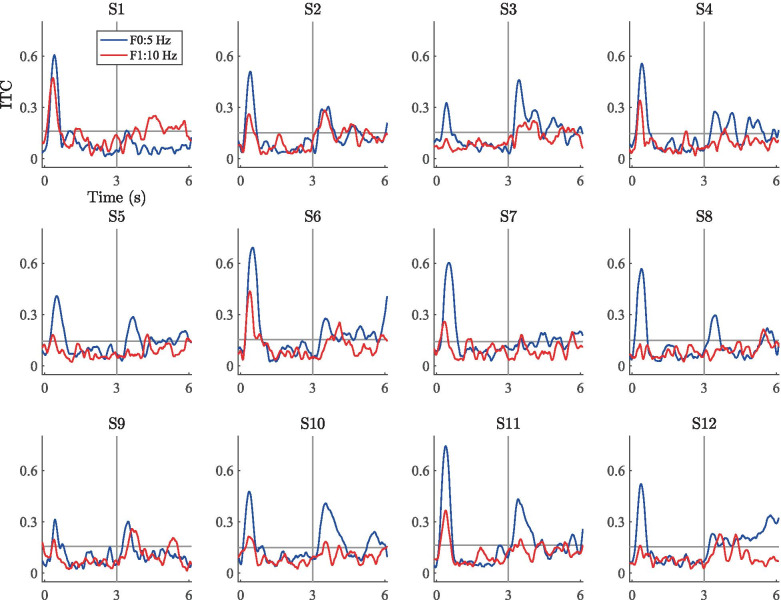
ITC time series for the 5-Hz wideband stimulus. Data presented as in Fig. 12

**Fig. 14:**
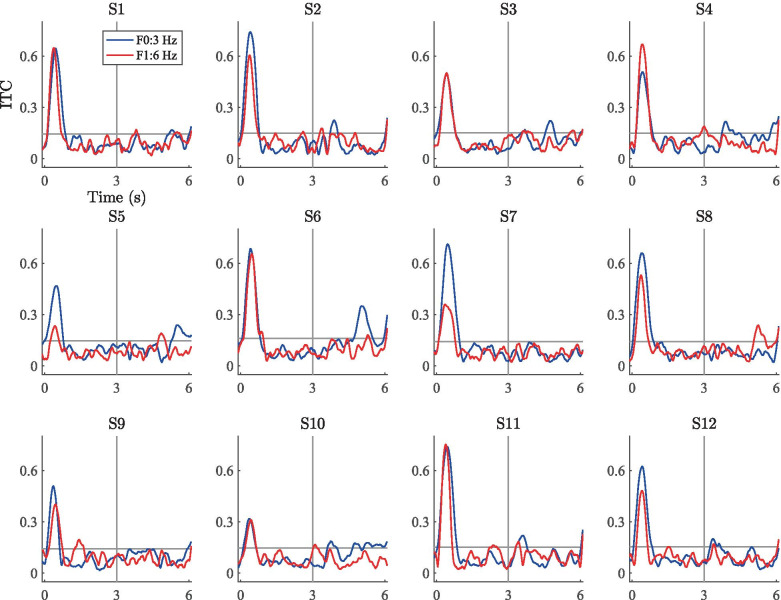
ITC time series for the 3-Hz ripple stimulus. Data presented as in Fig. 12

Figures 12, 13, and 14 show the subject-wise ITC-time series averaged across the fronto-central electrodes at the fundamental and first harmonic of the RCS modulations. Here, the highest ITC values are consistently seen at the stimulus onset (0-1 s) across subjects for each of the three stimuli. For most subjects, the introduction of the wideband RCS modulations at 3 s increased the ITC values during the latter half (3-6 s) of the stimulation above the subject-wise statistical thresholds given by Eq. (4). Generally, this ITC increase is most prominent at the onset of the wideband modulations (3 s in Figs. 12 and 13) and decays to a lower value during the latter half of the stimulation. Corresponding ITC increases for the ripple stimulus (Fig. 14) are much less prominent. Despite these general trends, inter-subject differences are also visible. For example, in the case of the 3-Hz wideband stimulus, Fig. 12 shows that while the responses from subject 2 display high ITC values during the modulated segment of the stimulation for both the fundamental and the first harmonic, the responses from subject 3 are qualitatively similar at the fundamental frequency but not at the first harmonic. In fact, the F1 ITC values for subject 3 are mostly below the subject-specific threshold of statistical significance throughout the entire modulated segment, while they are statistically significant for subject 2. ITC values for subject 1 on the other hand are below the statistical significance threshold for the majority of the modulated segment at both frequencies. Figure 13 shows the results from the 5-Hz wideband stimulus. Here, the inter-subject differences are similar to those observed for the 3-Hz wideband stimulus in Fig. 12, but the ITC values are generally lower during the modulation segments than for the 3-Hz wideband stimulus. There is nevertheless a noticeable increase in ITC between the two segments, as was confirmed by the group-level statistical analysis between the mean ITC values.

Overall, the ITC analyses show that the EEG responses across the fronto-central electrodes follow the RCS modulations in a phase-coherent manner for wideband stimuli, but not for the ripple stimulus. In general, ITC values appear to be significantly higher for the 3-Hz wideband stimulus than for the 5-Hz wideband stimulus, suggesting that low-frequency modulations yield more consistent EEG measures across repeated trials than high-frequency modulations.

## Discussion

In an attempt to improve the ecological validity of auditory scene analysis studies, recent neuroscientific experiments have employed novel synthetic stimuli that offer a balance between experimental control and spectro-temporal complexity (see Snyder and Elhilali (2017) for a review of recent developments). For example, several variants of the classic multi-tone masker paradigm (e.g., Neff and Green 1987; Neff and Callaghan 1988; Kidd et al. 1994, 2003) and the more recent stochastic-figure-ground paradigm (Teki et al. 2011; Teki et al. 2013) have been adopted in EEG, MEG and fMRI experiments, seeking to elucidate both the temporal dynamics and the neural bases of monaurally driven scene analysis processes (e.g., Micheyl et al. 2007; Gutschalk et al. 2008; Dykstra 2011; Elhilali et al. 2009b; Teki et al. 2011; Königs and Gutschalk 2012; Wiegand and Gutschalk 2012; Teki et al. 2013; Tóth et al. 2016). While these stimuli provide powerful tools for monaural scene analysis studies and take them a step closer to the spectro-temporal complexity that the auditory system faces in natural soundscapes, they are not well suited for studies targeting binaural processing in specific, since the salient grouping cues that they rely on are accessible under monaural listening. Currently, no binaurally driven stimulation paradigm has been established that could provide a satisfactory balance between spectro-temporal complexity and experimental control for neuroscientific scene analysis studies seeking to isolate binaural effects. Here, we sought to provide a potential solution to this methodological gap.

To this end, we used the RCS paradigm of Nassiri and Escabí (2008) to probe the EEG correlates of binaurally driven scene analysis processes. Specifically, we assessed the ERPs, change-onset responses, power spectra and inter-trial phase coherence of EEG responses to RCS stimuli consisting of an initial 3-s segment, where the envelopes of the binaural channels were uncorrelated, followed by another 3-s segment, where interaural envelope correlation was modulated according to the RCS paradigm. Our recordings show that in the case of wideband RCS modulations, the temporal dynamics of the obtained responses follow the ongoing modulations in a coherent manner both within and across trials. All aspects of our analyses (ERPs, PSD, ITC) indicate that EEG responses to wideband RCS modulations are more robust (i.e., yield larger ERP magnitudes as well as higher PSD and ITC values at the RCS modulation frequency and its harmonics) at the lower tested modulation rate of 3 Hz than at the higher modulation rate of 5 Hz. This observation is in accordance with previous EEG studies on modulated interaural coherence (Dajani and Picton 2006) as well as with the more general observation that the magnitudes of auditory ERPs increase with the inter-stimulus interval (Hocherman and Gilat 1981; Phillips et al. 1989; Bartlett and Wang 2005; Werner-Reiss et al. 2006; Brosch and Scheich 2008). Corresponding measures for RCS modulations shaped into a periodically repeating spectro-temporal ripple were much less prominent than those obtained for wideband modulations and generally failed to reach statistical significance.

Here, our stimulus selection sought to provide perceptually salient RCS stimuli. To that end, we picked the stimulus synthesis parameters (modulation frequency and ripple density) based on the psychophysical results reported in (Nassiri and Escabí 2008) as well as our subjective experiences with the stimuli. Our informal discussions with the subjects support the notion that both the wideband and ripple stimuli evoked the intended binaurally encoded percepts in a robust manner from trial-to-trial. Nevertheless, despite the perceptual salience of all three stimulus types, the EEG responses evoked by the ripple stimuli differ significantly from those evoked by wideband stimuli. Besides differences arising from the spectro-temporal activation patterns, the qualitative differences between the percepts evoked by the wideband and ripple stimuli could also have contributed the differences in the EEG responses. The wideband stimuli evoked perceptual phenomena that involved changes both in spatial perception and the number of perceived auditory objects. Namely, during the uncorrelated segments of the stimulation, the stimuli were perceived as two separate noise images at the two ears, while during the coherent segments, the percept switched to a single noise image at the auditory midline. In contrast, the introduction of the ripple-shaped RCS modulations did not result in a single fused image. Instead, two lateralized noise images remained at the two ears throughout the modulation cycle and a percept of a spectro-temporal ripple appeared. Therefore, the continuous frequency sweep percept evoked by the ripple stimulus did not involve periodic changes in the numerosity of perceived auditory objects or drastic changes in spatial perception during the modulation cycle. As such, the larger response magnitudes observed here with the wideband stimuli could be related to the fact the perceptual organization of the wideband stimuli changed in a more fundamental way during the RCS modulation cycle than it did for the ripple stimulus. This is an attractive hypothesis since changes in spatial perception are known to modulate cortical activity (e.g., Chait et al. 2005; Ross et al. 2007a, b) and the activity of the auditory cortex has been associated with object-level representations of auditory scenes (e.g., King et al. 2018).

In the present work, our aim was to evaluate the usability of the RCS paradigm in neuroscientific auditory scene analysis experiments. Accordingly, we restricted our stimuli to the two relatively simple RCS variants (wideband and spectro-temporal ripple) introduced in the original manuscript of Nassiri and Escabí (2008) to assess the cortical responses in an exploratory manner. However, the flexibility of the RCS paradigm lends itself to experimentation and could potentially enable the design of novel RCS stimulus variants that are better optimized to yield robust EEG correlates of binaural scene analysis than the basic RCS variants explored here. For example, Bardy et al. (2015) have shown that the magnitudes of auditory EEG responses depend on the (monaural) spectral complexity of the stimulation. Specifically, their study showed that line-spectrum stimuli (i.e., stimuli where low- and high-intensity spectral regions alternate across adjacent frequency bands) yielded higher magnitude EEG responses than flat-spectrum noise encompassing the same bandwidth (Bardy et al. 2015), supposedly due to decreased lateral inhibition. In the context of RCS stimuli, a binaurally coherent line spectrum could be created by limiting the coherent segments of the RCS modulations to non-adjacent spectral sub-bands, perhaps according to the bandwidths of auditory filters. Such a modification to the wideband modulations used here could potentially yield an increase in EEG response magnitude via decreased lateral inhibition. Further, the coherent sub-bands could be chosen cyclically across successive periods of the modulation to increase the temporal interval between binaurally coherent activations of individual spectral segments. This might increase the response magnitude simply by lengthening the inter-stimulation interval of the neurons activated by the binaurally coherent spectral segments in a frequency-specific manner.

Although binaural envelope correlation has received relatively little attention in previous scene analysis studies, its salience as a perceptual grouping cue is corroborated by recent theoretical accounts that emphasize the role of temporal coherence across sound features (e.g., amplitude envelope, pitch, spatial cues) in auditory perceptual organization (Elhilali and Shamma 2008; Shamma 2008; Elhilali et al. 2009a; Shamma and Micheyl 2010; McDermott et al. 2011; Shamma et al. 2011; Bizley and Cohen 2013; Micheyl et al. 2013; Shamma et al. 2013; Dykstra and Gutschalk 2013; Teki et al. 2013; Krishnan et al. 2014; O’Sullivan et al. 2015; Teki et al. 2016; Lu et al. 2017; King et al. 2018; Chakrabarty and Elhilali 2019). Common onsets and coherent amplitude modulation in particular have been shown to promote perceptual fusion of information carried in separate frequency channels into a unified auditory percept (Darwin 1997; Cusack and Carlyon 2003; Singh and Theunissen 2003; Elhilali and Shamma 2008; Shinn-Cunningham 2008; Elhilali et al. 2009a; Micheyl et al. 2013; Młynarski and McDermott 2019). The independent manipulation of the frequency-specific temporal fine structure and amplitude envelopes, offered by the RCS paradigm, allows the experimenter to leverage binaural envelope coherence-driven perceptual fusion, to promote perceptual grouping of frequency bands spanning the entire hearing range. This takes the RCS paradigm closer to ecological validity than what is achievable with IPD-based stimulation restricted to the low-frequency auditory channels, where fine structure timing information is available (Culling 1999). Therefore, the crucial advantage of the RCS paradigm over previously employed IPD-driven binaural stimuli (e.g., dichotic pitch or binaural beats) is that the perceptual organization of RCS stimuli is driven by the combination of two purely binaural signal features—interaural envelope coherence and fine-structure IPD—rather than by fine-structure IPD alone. As the steady-state responses observed here appear to be more prominent than those typically reported for classical binaural beat stimuli (e.g., Pratt et al. 2009, 2010) with beat frequencies similar to those of the RCS modulations used here, the increased spectro-temporal complexity allowed by the RCS paradigm may come with the secondary advantage of yielding more robust EEG responses.

Here, we used RCS stimuli with diotic fine structure, resulting in auditory images at the perceptual midline during the correlated segments of the stimulation. There is, however, no fundamental limitation to the stimulation paradigm that constrains the fused objects to the midline, and we see no reason why lateralization could not be incorporated as an experimental parameter by introducing frequency-specific and time-varying binaural cues to the stimulation. Influential accounts of auditory object formation (e.g., Woods and Colburn 1992) posit that the short-time-scale object formation process is driven primarily by non-spatial grouping cues and that directional percepts are formed according to the aggregate of the spatial cues contained in the frequency bands allocated to the same object. Indeed, there is a large body of research that suggests that spatial cues are relatively weakly weighted in auditory object formation (e.g., Assmann and Summerfield 1989, 1990; Shackleton and Meddis 1992; Bregman 1994; Culling and Summerfield 1995; Darwin 1997; Darwin and Hukin 1999; Shinn-Cunningham 2005; Darwin 2008; Schwartz et al. 2012), but yield a major advantage in facilitating auditory tasks unfolding across time, such as auditory streaming and speech perception in multi-talker scenes (Moore and Gockel 2012). Accordingly, we do not expect the presence of binaural cues within the naturally occurring range to have a significant effect on the auditory percepts evoked during the incoherent segments of RCS stimulation. Rather, the spatial interpretation of any interaural disparities embedded in the stimulus is expected to emerge only after a group of frequency bands have been bound together into a unified object during the coherent segments of the stimulation. Therefore, we find it plausible that the RCS paradigm could be expanded to include frequency-specific, as well as time-varying lateralization as an additional experimental parameter. This would further expand the range of stimuli offered by the paradigm but remains to be evaluated in future experiments.

Further research into the RCS paradigm and its variants could potentially enable future binaural scene analysis studies to be designed with more spectro-temporal flexibility than what has been possible with previously employed binaurally driven stimuli. Although time-varying versions of dichotic pitch stimuli allow the creation of binaurally encoded pitch sequences that also enable spatial manipulations (e.g., Dougherty et al. 1998), these stimuli still suffer the limitation of being necessarily restricted to the low-frequency range of IPD perception. Since binaural beat stimuli also rely on IPD, they have similar limitations. Furthermore, the spatial percepts of binaural beat stimuli are difficult to control as their generation mechanism involves on-going changes in interaural phase differences resulting from the interaction of detuned frequency components presented to the two ears. Although variants have recently been developed that yield larger EEG response magnitudes than typically observed with classical binaural beats and allow for discrete changes in spatial percepts (for instance, Ozdamar et al. 2011), these modified versions involve the use of precisely crafted modulations that introduce monaurally perceivable signal features into the stimulation. As such, these variants necessarily involve monaural confounds and are therefore not ideally suited for experiments seeking to isolate the neural correlates of binaural processes. Therefore, the RCS paradigm appears to offer some clear advantages over both dichotic pitch stimuli and binaural beats.

In the past, several studies have sought to identify neural correlates of scene analysis processes with noninvasive imaging techniques (e.g., Alain et al. 2002; Dyson and Alain 2004; Alain 2007; Bendixen et al. 2010; Tóth et al. 2016; Kocsis et al. 2016). An important line of this research has been to index the electromagnetic correlates of concurrent auditory object perception. Since the RCS paradigm can be leveraged to yield robust percepts of one or several concurrent auditory objects, we see it as a potentially useful stimulation paradigm for corroborating this line of auditory research. At the level of the ERP, multiple object perception is indexed by the object-related negativity (ORN) (Alain et al. 2001; Alain 2007), a systematic deviation in the deflection magnitudes of the N1 and P2 waves of the sound-onset response, relative to the ERPs associated with single-object percepts evoked by otherwise similar stimuli. ORN appears to originate from separate cortical substrates than those associated with the standard sound-onset complex (Arnott et al. 2011) and the fact that it can be recorded regardless of age or attentional state in human listeners (Alain and Izenberg 2003; Alain 2007; Alain and McDonald 2007; Folland et al. 2012; Bendixen et al. 2015) as well as in non-human primates (Fishman et al. 2014) suggests that it indexes a primitive scene analysis process operating independently of the cognitive state of the listener. Although ORN has been observed with a wide range of auditory stimuli and segregation-promoting cues (e.g., Alain et al. 2001, 2002; Johnson et al. 2003; McDonald and Alain 2005; Hautus and Johnson 2005; Sanders et al. 2008; Bendixen et al. 2010; Tóth et al. 2016; Kocsis et al. 2016), validating its appearance in response to RCS stimuli driven by binaural envelope coherence—a signal feature not previously assessed in ORN studies—would further elucidate the extent of its generality as a general electrophysiological marker of concurrent object perception.

Binaurally driven stimulation paradigms provide an especially attractive tool for studies seeking to disentangle the neural responses associated with auditory perceptual organization from those driven by the acoustic parameters of the stimulation. Under normal listening conditions, changes in perceptual organization are typically evoked by changes in acoustic parameters. In neuroscientific scene analysis experiments, this may make it difficult to reliably separate neural activations driven by acoustics from the activations associated with different aspects of perceptual organization or binaural processing. Stimuli such as the RCS provide the experimenter with a means of inducing changes in perceptual organization (e.g., numerosity of perceived objects) without the need to manipulate the acoustic parameters of the stimulus presented to either ear, thus effectively reducing the contributions of acoustically driven confounds to the neural responses. As such, RCS stimuli provide a promising paradigm for future investigations involving spectro-temporally complex auditory percepts without having to introduce a corresponding level of complexity into the acoustic parameters of the stimulation.
